# Prebiotic galactooligosaccharide feed modifies the chicken gut microbiota to efficiently clear *Salmonella*

**DOI:** 10.1128/msystems.00754-24

**Published:** 2024-07-31

**Authors:** Philip J. Richards, Abeer Almutrafy, Lu Liang, Geraldine M. Flaujac Lafontaine, Elizabeth King, Neville M. Fish, Amber J. Connerton, Phillippa L. Connerton, Ian F. Connerton

**Affiliations:** 1 Division of Microbiology, Brewing and Biotechnology, School of Biosciences, University of Nottingham, Loughborough, Leicestershire, United Kingdom; 2 Saputo Dairy UK (c/o Simon Hunt), Saputo Dairy UK Innovation Centre, Harper Adams University, Edgmond, Newport, United Kingdom; The University of Maine, Orono, Maine, USA

**Keywords:** prebiotic, GOS, galactooligosaccharide, chicken, *Salmonella*, SCFA, propionate, valerate, microbiome, microbiota

## Abstract

Chicken meat is contaminated with *Salmonella* from the gut of infected chickens during slaughter. Eradication of *Salmonella* from broiler chickens through hygiene measures and/or vaccination is not cost-effective; complementary approaches are required. A mature gut microbiota obstructs *Salmonella* infection in chickens, and deliberate fortification of colonization resistance through prebiotic feed formulations would benefit public health and poultry production. Prebiotic galactooligosaccharides hastens *Salmonella* clearance from the gut of infected chickens. To better understand the role of galactooligosaccharides in colonization resistance, broiler chickens were raised on a wheat-soybean meal-based feed, with or without galactooligosaccharides for the first 24 days of life. Chickens were orally challenged with *Salmonella enterica* serovar Enteritidis at 20 days and the effect of supplementary galactooligosaccharides characterized by profiling *Salmonella* colonization, gut microbiota, innate immune response, and cecal short-chain fatty acid concentrations. Exposure to dietary galactooligosaccharides shortened the time to clear *S*. Enteritidis from the ceca. Differential abundance analysis of the cecal microbiota associated *Salmonella* challenge with a bacterial taxon belonging to the Acidaminococcaceae family (*P* < 0.005). Increased cecal concentrations of the short-chain fatty acids propionate and valerate were measured in *Salmonella*-challenged chickens sustained on either control or galactooligosaccharide-supplemented feed relative to mock-challenged controls; but far greater concentrations were detected in chickens fed a galactooligosaccharide-supplemented diet in early life. The abundance of the Acidaminococcaceae taxon exhibited a positive correlation with the cecal concentrations of propionate (ρ = 0.724, *P* = 0.008) and valerate (ρ = 0.71, *P* = 0.013). The absence of cecal pro-inflammatory transcriptional responses suggest that the rapid *Salmonella* clearance observed for the galactooligosaccharide-supplemented diet was not linked to innate immune function.

**IMPORTANCE:**

Work presented here identifies bacterial taxa responsible for colonization resistance to *Salmonella* in broiler chickens. Deliberate cultivation of these taxa with prebiotic galactooligosaccharide has potential as a straight-forward, safe, and cost-effective intervention against *Salmonella*. We hypothesize that catabolism of galactooligosaccharide and its breakdown products by indigenous microorganisms colonizing the chicken gut produce excess levels of propionate. In the absence of gross inflammation, propionate is inimical to *Salmonella* and hastens intestinal clearance.

## INTRODUCTION

*Salmonella enterica* is a leading cause of diarrheal disease in humans (1), and *Salmonella enterica* subsp. *enterica* serovar Enteritidis in particular is a key causative agent of salmonellosis commonly associated with contaminated chicken meat and eggs (2, 3). Broiler chickens are birds specifically bred for meat production, and although vaccination of maternal broilers and stringent hygiene practices can exclude vertical transmission of *Salmonella*, factors such as short lifespan (typically 35–42 days) and cost make the vaccination of production broiler flocks unattractive to primary producers (4). Therefore, most broiler chickens raised in intensive farming systems are susceptible to *Salmonella* colonization through lapses in hygiene control, which includes introduction via contaminated feed or the entry of *Salmonella* from the outside environment (5). Husbandry practices that reduce the likelihood of flock colonization in the event of accidental *Salmonella* exposure are of interest to the poultry industry.

Colonization resistance is the exclusion of orally introduced organisms by the resident microbiota (6) and it has long been established that this phenomenon has a key role in protecting chickens from *Salmonella* colonization. In the absence of maternally inherited anti-*Salmonella* IgY (7), oral challenge of newly hatched chicks with *Salmonella* will effectively colonize the gastrointestinal tract with high densities of *Salmonella*. However, from ~7 days of age, a challenge of similar magnitude leads to less frequent successful colonization with lower levels of *Salmonella*, often without the overt presentation of disease symptoms (8–12). Colonization resistance can be deliberately produced in young chicks (<7 days old) through exposure to the gut microbiota of mature birds (12–14). However, hygiene interventions put in place in intensive production systems to prevent vertical pathogen transmission blocks exposure of chicks to the commensal and/or synergistic maternal gut microbiota, thereby potentially increasing the likelihood of gastrointestinal colonization by *Salmonella* (15).

Prebiotics are defined as substrates that are selectively utilized by microorganisms conferring a health benefit to the host (16). Deliberate modification of the chicken gut microbiota using prebiotics to bring about the gastrointestinal exclusion of *Salmonella* is desirable due to the relatively light regulatory burden they face and the ease of application relative to other feed supplements. Commercial prebiotic galactooligosaccharides (GOS) are enzymatically synthesized from lactose by β-galactosidase-catalyzed transgalactosylation, which produces β(1→3)-, β(1→4)-, and β(1→6)-linked galactose units with a terminal glucose as a mixture of oligosaccharides composed of two to eight monomeric units (17). GOS have been shown to accelerate the clearance of *Salmonella* Typhimurium from the ceca of layer hens (18). Parallel observations that the supplementation of drinking water with a GOS preparation led to increased cecal propionate levels in rats (19) and that competitive exclusion of *S*. Typhimurium in mice is mediated by propionate generated by *Bacteroides* (20) suggest a common mechanistic role for propionate.

Consumer demand for chicken meat remains high but competition between broiler chicken producers, in a market with rising costs of raw materials and energy, necessitates that production costs are tightly controlled. With the financial burden associated with dietary GOS inclusion in mind, supplementary GOS was included in the lower volume starter (0–10 days old) and grower (11–24 days old) diets at 2.32% (wt/wt) and 1.14% (wt/wt), respectively. We report that dietary GOS and *Salmonella* challenge shape the mature broiler chicken cecal microbiota to affect the abundances of short-chain fatty acids (SCFAs), which act to efficiently clear *Salmonella* colonization.

## RESULTS

### Dietary GOS in early life hastens *Salmonella* cecal clearance from broiler chickens

**Fig 1:**
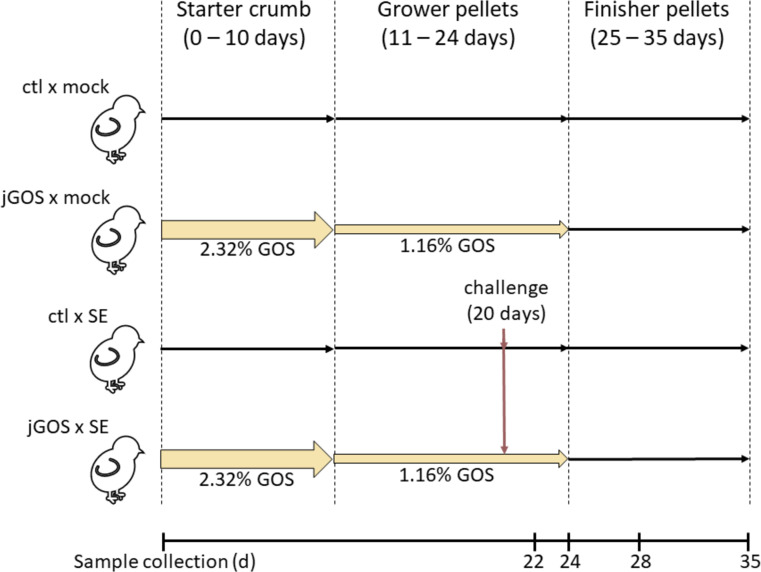
Trial design. A total of 112 chickens were placed in 28 pens of 4 evenly divided between 4 treatment groups (ctl × mock, control diet with mock *S*. Enteritidis colonization; ctl × SE, control diet with *S*. Enteritidis colonization; jGOS × mock, GOS supplemented diet with mock *S*. Enteritidis colonization; jGOS × SE, GOS supplemented diet with *S*. Enteritidis colonization). Samples were collected by culling a single bird collected from each pen at each experimental timepoint.

Azcarate-Peril et al. (18) reported that supplementation of a corn-soybean-based feed with 0.55% (wt/wt) GOS hastened clearance of *S*. Typhimurium from the ceca of white leghorn layer chickens. However, the effectiveness of prebiotics in poultry is dependent on the prebiotic formulation and the microbes colonizing the gastrointestinal tract (21). To test if GOS promoted the clearance of *Salmonella* from broiler chickens sustained on an alternate standard feed composition, we orally challenged 20-day-old Ross 308 broiler chickens raised on wheat-soybean meal-based feed with *Salmonella* Enteritidis. Chickens were fed either a control diet (ctl group) or a diet with supplementary GOS inclusion for juvenile birds up until 24 days old (jGOS group). As age is known to influence both the persistence of *Salmonella* infection and the ecology of the gut microbiota (11, 22, 23), intestinal contents and tissue samples were collected from birds sacrificed over a period of 15 days post-infection (dpi) with the endpoint at 35 days being a commonplace slaughter age. Therefore, *Salmonella* colonization levels are reported with respect to both the age of the birds and the time duration post challenge as summarised in Fig. 1. No *Salmonella* were recovered from the intestinal contents or organs of the mock-challenged chickens fed either the control or the GOS-supplemented diet.

**Fig 2:**
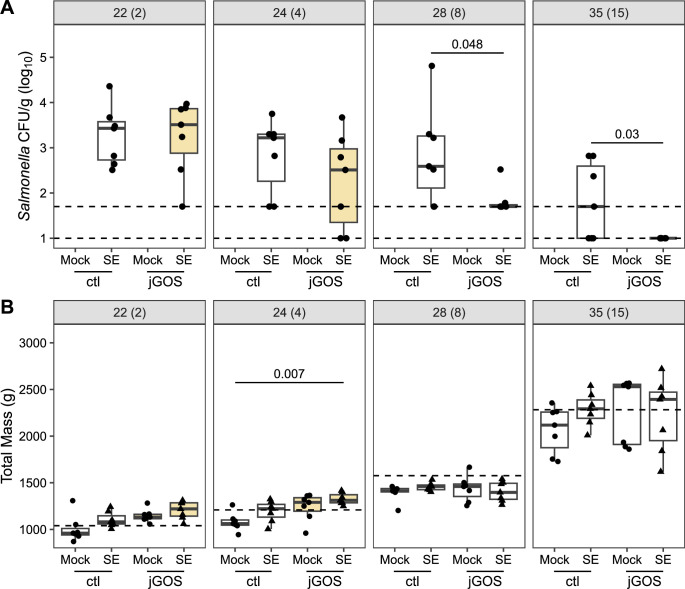
GOS-supplemented feed in early life hastens the clearance of *S.* Enteritidis from chickens. Birds were challenged with *S*. Enteritidis at 20 days of age. The fill color indicates the diet: unfilled, control feed without GOS; yellow, feed supplemented with GOS. The shaded strip areas above each panel indicate the ages of the bird in days followed by days post-infection in parentheses. (**A**) Cecal burden of *S*. Enteritidis. Each data marker represents an individual cecal community/bird collected from each independent pen (*n* = 7). The number above the bar indicates *P*-value, determined using Wilcoxon rank sum test. Only *P*-values <0.05 are shown. The upper dashed line indicates limit of direct enumeration, the lower dashed line indicates limit of detection by enrichment. (**B**) Chicken body weight for each of seven pens. Significance was determined using the Kruskal-Wallis test for body weights measured at each timepoint. For those ages with a *P*-value below a significance threshold of 0.05, a subsequent pairwise Wilcox test with Benjamini-Hochberg adjustment for multiple comparisons was performed to determine significance between cohorts. The number above the bar indicates the adjusted *P*-value. Only adjusted *P*-values <0.05 are shown. The dashed line indicates breed performance objective for male Ross 308 chickens (2014).

At 22 and 24 days old, equal to 2 and 4 dpi, similar levels of viable *Salmonella* were cultured from the cecal contents of *Salmonella*-challenged chickens raised on either control or GOS-supplemented feed (*P ≥* 0.198; Fig. 2A). However, by 28 days old (8 dpi), the levels of *Salmonella* isolated from the cecal contents of chickens on the control diet were greater than those recovered from jGOS birds (*P* = 0.048). Correspondingly, *Salmonella* could only be directly enumerated from two of the seven chickens from the jGOS group, with recovery of *Salmonella* from the cecal content of the remaining five birds dependent on enrichment culture methods. *Salmonella* was directly enumerated from five of the seven chickens on the control diet, with enrichment only required to recover *Salmonella* from two birds, indicating that higher levels of *Salmonella* were present in chickens raised on the control feed (Fig. 2A). One week later, at 35 days old (15 dpi), the levels of *Salmonella* isolated from chickens from the jGOS group remained lower than those in chickens on the control diet (*P* = 0.03). At this time, *Salmonella* was directly enumerated from three of the seven chickens on the control diet but was not recoverable from the cecal contents of any of the seven chickens from the jGOS group even with the aid of enrichment (Fig. 2A). *S*. Enteritidis is frequently found in the spleen and liver of chickens after oral challenge (24). By 4 dpi, *Salmonella* was detected by enrichment from the spleens and livers of 7 of 7 birds from the challenged chickens of both the control and jGOS diet groups, but by 15 dpi (35 days old), *Salmonella* could only be enriched from the livers of two of seven chickens in each group (Table S1). Correspondingly, *Salmonella* was recovered from the spleens of three of seven chickens from the control group and two of seven chickens from the jGOS group.

Previously, we have reported that supplementing feed with GOS improves the growth rate of *Salmonella*-free chickens on a wheat-soybean meal-based feed (25). In the results presented here, the body weights of *Salmonella*-challenged chickens in the jGOS group were greater than mock-challenged chickens on the calorie-balanced control diet at 24 days old (*P* = 0.007; Fig. 2B), corresponding with the end of the period during which supplementary GOS was added to the diet. No significant difference in the body weights was detected at 28 or 35 days, which included the *Salmonella* Enteritidis (SE)-challenged birds that did not suffer any growth check. These findings indicate that while dietary inclusion of GOS in early life hastened the clearance of *Salmonella*, the inclusion did not effect the body weight at 35 days old.

### Dietary GOS in early life affects cecal microbiota diversity following *Salmonella* Enteritidis challenge

**Fig 3:**
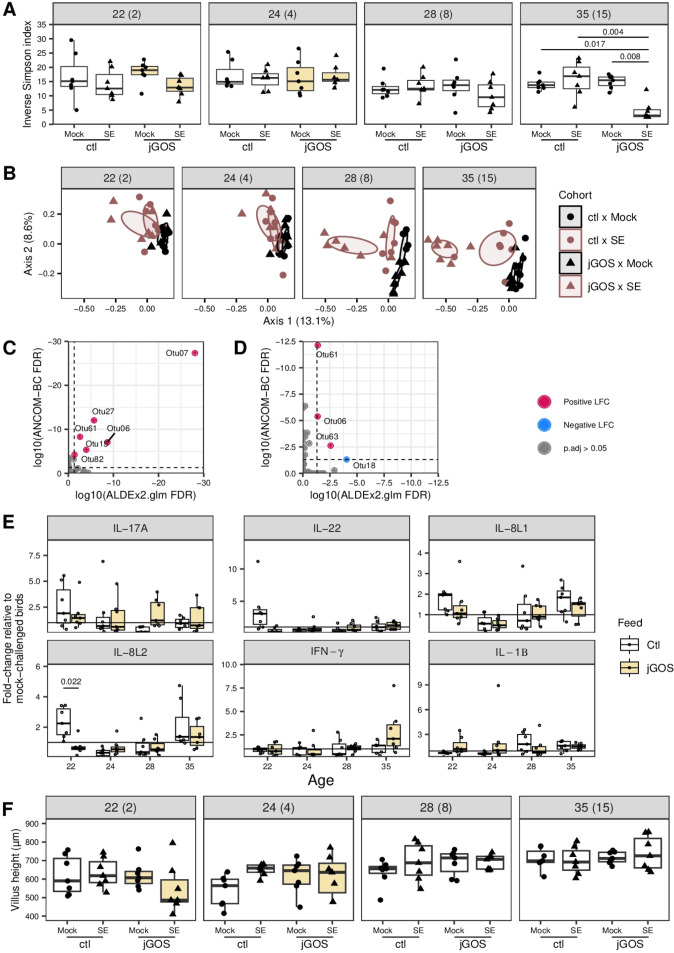
A distinct microbiota distinguishes *S.* Enteritidis-challenged chickens raised on a GOS-supplemented diet. (**A**) α-Diversity as represented by inverse Simpson index. Each data marker represents individual cecal community/bird collected from each independent pen (*n* = 7). The shaded strip areas indicate the bird age in days followed by days post-infection in parentheses. Statistical significance was determined using the Kruskal-Wallis test for inverse Simpson indices measured at each timepoint; for those timepoints with a *P*-value below a significance threshold of 0.05, a Dunn’s test of multiple comparisons with Benjamini-Hochberg adjustment was performed to determine significance between cohorts. The number above the bar indicates *P*-value. Only *P*-values <0.05 are shown. The fill color indicates the diet at the time of sampling: unfilled, control feed without GOS; yellow, GOS-supplemented feed. (**B**) Principal component analysis of the Bray-Curtis dissimilarities (*r*^2^ = 0.55%). Each data marker represents an individual cecal community/bird collected from each independent pen (*n* = 7). Shaded eclipses indicate 95% confidence levels calculated using ggpubr (27) as per the FactoMineR package (28). For each cohort (ctl is the control diet, and jGOS is the juvenile GOS-supplemented diet) with those shaded gray mock colonized and those shaded red colonized with *S*. Enteritidis. The shaded strip areas above each panel indicate the ages of the bird in days followed by days post-infection in parentheses. The sequencing error rate was determined as 0.14% using a control cocktail of eight bacterial species (sum of mismatches to reference / sum of bases in query). (**C**) Discriminative taxa in SE-challenged chickens identified using multivariate analysis in both ALDEx2 (version 1.30.0) and ANCOM-BC (version 2.0.1) packages. Superfluous “0”s in operational taxonomic unit (OTU) ID have been removed for clarity. OTUs were identified as follows: Otu06, *Megamonas* (Otu0006); Otu07, Acidaminococcaceae_unclassified (Otu0007); Otu15, *Bifidobacterium* (Otu0015); Otu27, *Bacteroides* (Otu0027); Otu61, *Lactobacillus* (Otu0061); Otu82, *Olsenella* (Otu0082). (**D**) Discriminative taxa in jGOS-raised chickens identified using multivariate analysis in both ALDEx2 and ANCOM-BC packages. Superfluous “0”s in OTU ID have been removed for clarity. OTUs are identified as follows: Otu06, *Megamonas* (Otu0006); Otu18, Ruminococcaceae_unclassified (Otu0018); Otu61, *Lactobacillus* (Otu0061); Otu63, *Enterococcus* (Otu0063). (**E**) Effect of *S.* Enteritidis challenge on expression of cytokines IL-17A, IL-22, IL-8L1, IL-8L2, IFN-γ, and IL-1β in the ceca. Gene expression calculated as 2^-(ΔΔCt)^ and presented as fold change in *Salmonella*-challenged chickens relative to the mean expression level of mock-challenged chickens for each feed group (control or jGOS). Note that *n* = 7 for all groups, excluding the control diet, mock-challenged cohort value for IL-17A at 22 days old (*n* = 7). The number above the bar indicates the adjusted *P*-value as determined using Student’s *t*-test for each pair of expression comparisons per gene per timepoint with false discovery rate correction performed across each timepoint using the Benjamini-Hochberg method. Only *P*-values <0.05 are shown. Shaded strips above each panel indicate the corresponding gene name. (**F**) Ileal villus length per individual bird collected from each independent pen (*n* = 7). Statistical significance was determined using Tukey’s Honest Significant Difference method with adjustment for the multiple comparisons per timepoint. No *P*-values are shown on the figure as all were in excess of a significance threshold of 0.05.

Mon et al. (26) reported that *S*. Enteritidis colonization of the cecum of 2-week-old layer chickens was accompanied by changes in the bacterial microbiota without any indication of disease. We were interested to know if the cecal microbiota of broiler chickens was similarly affected by *Salmonella* challenge and whether supplementary GOS in early life might disrupt these changes and potentially affect *Salmonella* clearance. The cecal microbiota of the experimental chickens was profiled using 16S rRNA amplicon sequencing to an adequate sequencing depth (Fig. S1). In agreement with the data of Mon et al. (26), we observed that whereas the α-diversity of the cecal community of chickens sustained on the control feed was unchanged by SE challenge (inverse Simpson index: *P* ≥ 0.682, Fig. 3A; Shannon entropy: *P* ≥ 0.697, Fig. S2), changes the β-diversity of these bacterial communities did accompany *Salmonella* colonization, as revealed by analysis of the Bray-Curtis dissimilarity (Fig. 3B). However, when supplementary GOS was introduced to the diet of juvenile chickens, by 15 dpi, the α-diversity of the bacterial communities in the ceca was significantly lower in SE-challenged birds than that of all other treatment groups (inverse Simpson index: *P* ≤ 0.017, Fig. 3A; Shannon entropy: *P* ≤ 0.012, Fig. S2). Correspondingly, chickens on the jGOS feeding regime also exhibited a more profound change in the β-diversity of the cecal microbiota following SE challenge than mock-challenged chickens from the jGOS group, and both the SE- and mock-challenged chickens in the control feed group (Fig. 3A). Changes in β-diversity are particularly evident from 8 dpi onward. These data show that sustaining broiler chickens on a GOS-supplemented diet up until they are 24 days old affects changes in the cecal microbiota caused by *Salmonella* challenge 10 days after supplementary GOS was withdrawn.

By comparison with other forms of analysis, Nearing et al. (29) showed that ALDEx2 (30–32) and ANCOM-based approaches (33, 34) delivered the most conservative results for the identification of discriminative taxa, with a lower risk of false positives. Confidence that the operational taxonomic units (OTUs) identified by the procedure were genuine and not the result of bacterial DNA contamination of the kit or reagents was evident from their absence or low abundance in the “kitome” negative control (Table S2). ALDEx2 and ANCOM-based packages were used here to determine those OTUs associated with either SE challenge or inclusion of supplementary GOS; the greatest *P*-value returned by either package is reported here. SE challenge resulted in the increased abundance of six bacterial OTUs, and in particular, Acidaminococcaceae_unclassified (log_10_ fold change = 6.86, *P*-value < 0.005). The remaining OTUs that were positively associated with SE challenge but with smaller effect size were as follows: *Megamonas* spp. (Otu0006; log_10_ fold change = 3.42, *P*-value < 0.005), *Bifidobacterium* spp. (Otu0015; log_10_ fold change = 3, *P*-value < 0.005), *Bacteroides* spp. (Otu0027; log_10_ fold change = 4.54, *P*-value < 0.005), *Lactobacillus* spp. (Otu0061; log_10_ fold change = 2.71, *P*-value ≤ 0.002), and *Olsenella* spp. (Otu0082; log_10_ fold change = 2.08, *P*-value ≤ 0.041) (Fig. 3C). No OTUs showed significant reductions in abundance in response to SE challenge.

Across all timepoints, the abundance of three taxa exhibited a positive log fold change (FC) in response to the jGOS feeding regime: *Megamonas* spp. (Otu0006; log_10_ fold change = 2.99; *P*-value ≤ 0.043), *Lactobacillus* spp. (Otu0061; log_10_ fold change = 3.69; *P*-value ≤ 0.042), and *Enterococcus* spp. (Otu0063; log_10_ fold change = 1.05; *P*-value ≤ 0.003) (Fig. 3D). Both *Megamonas* spp. (Otu0006) and *Lactobacillus* spp. (Otu0061) were increased in chickens on the jGOS diet while also showing association with SE challenge. Ruminococcaceae_unclassified spp. (Otu0018; log_10_ fold change = −1.38; *P*-value ≤ 0.049) was the sole taxa reduced by the jGOS diet (Fig. 3D). In contrast to the blooms in the relative abundance of bacteria of the phylum Proteobacteria reported to accompany *Salmonella*-mediated inflammation in mouse infection models (35), no taxa classified as Proteobacteria were observed to increase in abundance in response to *Salmonella* challenge in the chicken intestinal microbiota (Fig. 3C), possibly due to the absence of gross inflammation.

*Salmonella* colonization and clearance in chickens are linked to innate and adaptive immunological responses. Innate immune responses evident in, but not exclusively, the ileum and ceca are reported to involve IL-17, IL-22, IL-8, IFN-γ, and IL-1β (36–38). We therefore profiled the expression of a panel of these and other immune function-linked genes in cecal tissues to determine if the influence of GOS on the microbiota was associated with changes in host immunity. The supplementation of juvenile feed with GOS differentiated the host response of chickens to SE colonization in the cecum at 22 days old (2 dpi), with up-regulation of IL-8L2 (*P* = 0.022) observed in SE-challenged birds on the control diet (Fig. 3E), alongside up-regulation of STAT3 (*P* = 0.022) and TGIF1 (*P* = 0.017) (Fig. S3).

Increases in anti-*Salmonella* serum antibodies were detected by enzyme-linked immunosorbent assay (ELISA) in response to *Salmonella* challenge. *Salmonella*-infected chickens exhibited an increase in IgY compared to the non-challenged birds at 8 dpi (control diet *P* = 0.049; jGOS-supplemented diet *P* = 0.042) and increases for IgY and IgA at 15 dpi (control diet IgY *P* = 0.024 and IgA *P* = 0.008; jGOS-supplemented diet IgY *P* = 0.004 and IgA *P* = 0.006). No significant differences were observed between *Salmonella*-infected chickens on the control and jGOS-supplemented diets for either IgY or IgA at any of the sampling timepoints (Fig. S4).

Diarrhea and mild inflammation of the ileum wall are associated with *Salmonella* infection of chickens (36). In the study reported here, chickens challenged at 20 days old did not exhibit any change in behavior or clinical signs associated with salmonellosis in chickens or exhibit any gross pathology post-mortem. Hematoxylin and eosin-stained histological sections of ileal tissues revealed no significant differences in villus height at any timepoint (*P^_adj_^* ≥ 0.086, Fig. 3F). However, we note that *Salmonella* could be detected by enrichment in the spleens and livers of infected birds at 2, 4, 8, and 15 dpi (Table S1), but with minimal mononuclear cell infiltration of the hepatic parenchyma evident. The incidence of *Salmonella* recoverable from the liver was reduced to two birds from each group by 15 dpi (35 days old) with no observable pathology.

### Cecal SCFA concentrations are correlated with Acidaminococcaceae

**Fig 4:**
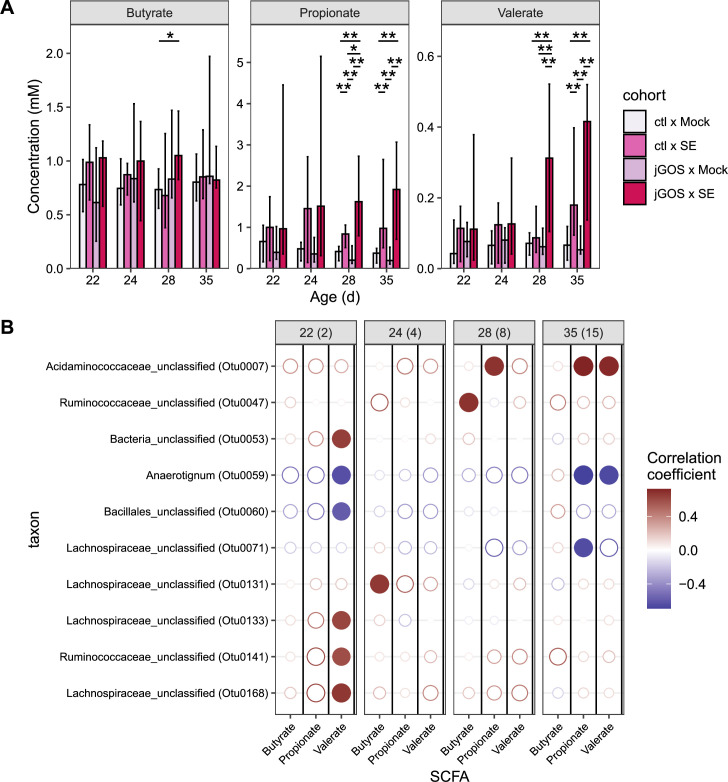
Relationship of microbiota to SCFA profile. (**A**) Concentration of butyrate, propionate, and valerate in the cecal lumen. The plot shows the median concentration of seven, six individual cecal community/bird collected from each independent pen, apart from ctl × mock at 35 days (*n* = 6) and jGOS × SE at 22 and 35 days (*n* = 6) due to difficulties in extracting the sample. Plot error bars indicate the upper (97.5th) and lower (2.5th) percentiles. Statistical significance was determined using the Kruskal-Wallis test for each SCFA measured at each timepoint; for those SCFA with a *P*-value below a significance threshold of 0.05, a subsequent pairwise Wilcox test with false discovery rate (FDR) correction was performed to determine significance between groups. Asterisk symbol above the bar indicates pairwise Wilcox test *P*-value. For clarity, significance is indicated by an asterisk above bar: *P* < 0.001, ***; *P* < 0.01, **; *P* < 0.05, *. (**B**) Correlation plot showing significant relationships between bias-corrected OTU abundances and absolute concentration (ppm) of the SCFA metabolome. Both size and color of the data marker indicate Spearman correlation coefficient with FDR correction for each SCFA at each timepoint. Filled data markers indicate significance (*P* ≤ 0.05). Results are reported at OTU taxonomic level. Due to failure to measure SCFA in some samples, outlined above, the correlations were calculated on the basis of 27 measurements at 22 days, 28 measurements at 24, 28 measurements at 28 days, and 26 measurements at 35 days.

SCFAs are produced in the gut through the microbial breakdown of carbohydrates (39), and the antimicrobial potential of SCFAs in the chicken gut has been recognized for some time (40). Given that supplementation of drinking water with a GOS preparation increases cecal propionate levels in rats (19) and that increased levels of gastrointestinal propionate have been established as protecting against *Salmonella* infection in mice (41), we investigated whether shifts in SCFA were associated with provision of GOS-supplemented feed in early life and/or *Salmonella* challenge of broiler chickens. Concentrations of propionate and eight other SCFAs (acetic acid, isobutyric acid, butyrate, 2-methyl butyric acid, isovaleric acid, valerate, hexanoic acid, and lactic acid) were measured in luminal cecal contents, but significant differences in SCFA levels between cohorts were only evident for propionate, butyrate, and valerate. A similar trend was noted for each of these SCFAs with greater concentrations detected in *Salmonella*-challenged chickens, and most profoundly in *Salmonella*-challenged chickens in the jGOS group. At 8 dpi (28 days old), butyrate levels in SE-challenged, jGOS group chickens (mean 1.1 mM) were greater than unchallenged chickens raised on control feed (mean 0.7 mM; *P* = 0.024; Figure 4A), but at this time, all groups were better differentiated by propionate and valerate concentrations which were significantly higher in SE-challenged chickens in the jGOS group than any other group (propionate *P* ≤ 0.013; valerate *P* ≤ 0.008). At 35 days old, the concentrations of propionate and valerate were greater in the SE-challenged chickens in the jGOS group than mock-challenged chickens on the same dietary regimen (propionate *P* ≤ 0.003; valerate *P* ≤ 0.007). Correspondingly, the levels of propionate measured for the SE-challenged chickens on the control diet exceeded those for the mock-challenged chickens on the control diet at 28 and 35 days (*P* ≤ 0.006 and *P* ≤ 0.003, respectively), and the levels of valerate were greater in SE-challenged chickens on the control diet than mock-challenged chickens on the control diet at 35 days (*P* ≤ 0.007). Notably, propionate was also detected at greater levels in SE-challenged chickens sustained on the control diet than the mock-challenged jGOS chickens at 28 and 35 days, and similarly, valerate concentrations were greater in the SE-challenged chickens on the control diet than the mock-challenged jGOS group at 35 days (*P* ≤ 0.007). These observations suggest that the increased levels of cecal propionate and valerate are a consequence of the SE challenge, but greater concentrations of these SCFAs can be achieved in chickens belonging to the jGOS group.

A correlation analysis was undertaken on the observations of the levels of propionate, butyrate, and valerate and the abundance of the OTUs from the ceca of each individual chicken. Due to the recognized drawbacks inherent in proportion-based approaches (42), bias-corrected OTU abundances were generated using the ANCOM-BC package and the association between these abundances and the concentration of each SCFA was calculated by Spearman’s rank coefficient (ρ). Although significant negative correlations were plentiful, only seven taxa had significant positive correlations with either butyrate, propionate, or valerate (Fig. 4B). Acidaminococcaceae_unclassified (OTU0007) was correlated with propionate at 28 (ρ = 0.68, *P* = 0.018) and 35 days (ρ = 0.724, *P* = 0.008). Acidaminococcaceae_unclassified (OTU0007) was also positively correlated with valerate at 35 days (ρ = 0.71, *P* = 0.013). Ruminococcacea_unclassified spp. (OTU0047) was positively correlated with butyrate at 28 days (ρ = 0.68, *P* = 0.018).

Two OTUs were negatively correlated with propionate at 15 dpi: *Anaerotignum* (Otu0059) (ρ = −0.691, *P* = 0.012) and Lachnospiraceae_unclassified (Otu0071) (ρ = −0.651, *P* = 0.025), both of which belong to the Lachnospiraceae family. *Anaerotignum* spp. (Otu0059) was also negatively correlated with valerate at 2 dpi (ρ = −0.636, *P* = 0.029) as was Bacillales_unclassified (Otu0060) (ρ = −0.59, *P* = 0.042). Acidaminococcaceae_unclassified (Otu0007) was positively correlated with propionate and valerate concentration and was increased in SE-challenged birds. No overlap was observed between propionate-, butyrate-, and valerate-correlated OTUs and taxa associated with the jGOS group.

Correlation analysis was also performed using OTU relative abundance (%) in place of bias-corrected OTU abundances (Fig. S5). This approach identified 34 OTUs with significant positive or negative associations with either butyrate, propionate, or valerate, compared with a total of 10 OTUs identified using the previous approach. However, there was agreement between the approaches with seven OTUs identified as having similar significant correlations. Of the six taxa found to be positively associated with SE challenge using the ALDEx2 and ANCOM-based approaches, all were positively correlated with propionate or valerate at some stage. Similarly, of the three taxa increased in the jGOS group, *Megamonas* spp. (Otu0006) and *Lactobacillus* spp. (Otu0061) were positively correlated with propionate and valerate. However, Ruminococcaceae_unclassified (Otu0018) that was negatively associated with the jGOS diet showed no correlation with either butyrate, propionate, or valerate.

## DISCUSSION

We have shown that supplementing the feed of juvenile chickens with GOS until the birds are 24 days old shortens the time required for clearance of *S*. Enteritidis infection following an oral challenge at 20 days. Oral gavage with *Salmonella* Enteritidis did not result in overt intestinal disease, as characterized by intestinal inflammation or reduced growth performance, but did generate increased concentrations of cecal propionate at 8 and 15 dpi in chickens raised on either control or jGOS diets relative to diet-matched mock-challenged controls. At 15 dpi, the levels of propionate detected in *Salmonella*-challenged chickens were greater in chickens in the jGOS group (1.98 mM) relative to *Salmonella*-challenged chickens on the control diet (1.17 mM), establishing a link between SE challenge and cecal propionate that is augmented by, but not dependent on, juvenile exposure to supplementary GOS. In parallel, valerate concentrations were observed to be greater in the *Salmonella*-challenged jGOS group (0.38 mM) compared to *Salmonella*-challenged chickens on the control diet (0.22 mM). As supplementary GOS was absent from the diet from 24 days old (4 dpi), this is likely a legacy effect of consuming GOS. Culture-based experiments have ascertained that 15 mM propionate at pH 6.5 will inhibit the growth of *S*. Typhimurium (43); whereas 25 mM propionate at pH 6 is required to inhibit the growth of *S*. Enteritidis (44). Moreover, 3.13 mM propionate is reported to increase the lag time of *S*. Typhimurium and 6.25 mM propionate to decrease the growth rate (20). Valerate is also inimical to *Salmonella* in culture with inhibitory concentrations for *S*. Typhimurium and *S*. Enteritidis reported between 25 and 37 mM (45, 46). Although the concentrations of the SCFAs we measured in the cecal contents are lower than required *in vitro*, it is possible that the inimical effect of propionate combined with valerate is heightened at the pH of the ceca or the niches occupied by *Salmonella*.

Although propionate and butyrate are reported to have immunoregulatory activities in mice (47), we did not find evidence of any host-mediated responses characteristic for chickens in the jGOS group, and therefore, the direct action of the SCFAs propionate and valerate on *Salmonella* seems more likely. The mechanism by which propionate inhibits *Salmonella* growth has yet to be established, but the predominant hypothesis is that dissociated propionate diffuses across the cell membrane and acidifies the cytoplasm (20, 40, 48). The alternate and possibly complementary hypothesis is that at 10 mM, propionate represses *S*. Typhimurium invasion through post-translational control of HilD (49). However, an extensive study by Shelton et al. (50) showed that in the inflamed mouse gut, *S*. Typhimurium can break down propionate into pyruvate via the 2-methylcitric acid cycle, which is encoded by the *prpBCDE* operon (51). Studies using *S*. Typhimurium have indicated that activation of the *prpBCDE* operon is dependent on the availability of respiratory electron acceptors, such as oxygen in aerobic respiration (52) or nitrate during anaerobic respiration (50). While *S*. Enteritidis P125109 has an intact *prpBCDE* operon that can in principle metabolize propionate (51, 53), the chicken cecal lumen maintains a flourishing anaerobic community due to the avid consumption of capillary oxygen by the enterocytes catabolizing butyrate generated by the microbiota, and in the absence of an inflammatory response to *S*. Enteritidis, the electron acceptor nitrate will not be available to activate *prpBCDE* gene expression. Under these circumstances, *S*. Enteritidis would not be able to prevent propionate toxicity.

In mice, the prohibitory effect of luminal propionate on *Salmonella* is dependent on the immune response of the host since although propionate mediates colonization resistance to *S*. Typhimurium in conventionally colonized mice (20), an extensive study by Shelton et al. (50) showed that following streptomycin treatment, *Salmonella* challenge resulted in inflammation in the gut, and under these conditions, propionate can act as a carbon source for *S*. Typhimurium. Parallels of this can be recognized in the literature reporting *Salmonella* colonization of chickens, while being aware of the established differences in virulence between *Salmonella* serovars (54), virulence factor-induced inflammation of 1-day-old chicks has been shown to facilitate the outgrowth of *S*. Enteritidis by aerobic respiration (55) and a profile of *S*. Typhimurium gene expression in the cecum of chicks challenged at 12 h from hatch identified propionate as a carbon source (56). A comparative genomic study of gastrointestinal and extraintestinal pathovars of *Salmonella* revealed the extraintestinal pathovars to have degraded the regulators for anaerobic processes including *prpR*—the propionate catabolism operon regulatory protein (57). The authors conclude the retention of these regulatory networks provides the gastrointestinal pathovars the opportunity to outcompete other microorganisms present in the lumen of the inflamed gut. However, the involvement of propionate in infection is likely complex since *Salmonella* Gallinarum is an extraintestinal pathovar (the causative agent of systemic fowl typhoid in chickens) that retains the *prpR* gene but does not trigger a pro-inflammatory response in the gut, and is reported to be less likely to cause disease in chickens sustained on feed supplemented with propionate (41, 53, 58, 59). However, micro-encapsulated dietary propionate did not afford protection to young chickens (5 days) exposed to *S*. Enteritidis that also possess the *prpR* gene (60). Levels of valerate followed a similar trend to propionate and were also higher in *Salmonella*-challenged chickens, especially in the presence of GOS that led to the clearance of intestinal *S*. Enteritidis. Consistent with this observation, chickens with low heterophil/lymphocyte ratios show resistance to *S*. Enteritidis and where the lower abundances of *S*. Enteritidis are reported to correlate with greater cecal propionate and valerate contents (61). There are, however, few reports of the origin of microbiota-generated valerate in the literature, nor of its effect on the host. Nevertheless, valerate has been shown to have anti-*Clostridium difficile* activity in mice (62). No OTUs from the *Clostridium* cluster XI were found to discriminate between cohorts in this study, but *Anaerotignum* spp. (Otu0059) which falls within the broader grouping of the class Clostridia were negatively correlated with both cecal propionate and valerate concentrations. We observed that *Salmonella* was more effectively cleared from the cecal pouches of chickens from the jGOS group, but that the organism persisted in the liver and spleen, and potentially other extraintestinal tissues. Selective clearance of intestinal *Salmonella* with GOS, possibly in combination with other non-antibiotic feed additives (63) may be exploited in future studies to determine the potential for the persistence and re-colonization of the gut by extraintestinal cells.

Amplicon profiling indicated that Acidaminococcaceae_unclassified (Otu0007) was associated with *Salmonella* challenge and correlated with the levels of cecal propionate and valerate. Querying the representative OTU sequence for Otu0007 using NCBI BLASTn (64) revealed similarity to *Phascolarctobacterium faecium* (E-value = 6e−110; % identity = 96.03%; accessed 3 March 2024). *P. faecium* has been shown to produce propionate through the catabolism of the dicarboxylic acid succinate (65). Experiments using *in vitro* batch fermentation have shown that GOS is fermented to succinate by the human microbiota (66), suggesting that the increased levels of *P. faecium* are due to cross-feeding and not directly the catabolism of GOS. *Megamonas* spp. (Otu0006) was also associated with SE challenge, and a positive correlation was shown between its proportional abundance and the cecal concentrations of propionate and valerate. *Megamonas* spp. are also known to metabolize succinate and produce propionate (43, 67–69), and chickens dosed with a probiotic *Bacillus* cocktail (two *Bacillus subtilis* strains, plus *Bacillus amyloliquefaciens*) were reported to produce significant increases in fecal propionate and in the abundance of *Megamonas* (70). Acidaminococcaceae_unclassified (Otu0007) and *Megamonas* spp. (Otu0006) are closely related taxa. The representative OTU sequences from this trial have 86.22% sequence identity, and on the basis of 16S rRNA gene sequence alignment, it has been reasoned that the genus *Megamonas* be reclassified as a member of the family Acidaminococcaceae (71). However, if the Acidaminococcaceae_unclassified (Otu0007) and *Megamonas* spp. (Otu0006) similarly benefit from the provision of substrate succinate, this leaves the identities of the primary GOS degraders still to be determined. The 16S rRNA sequencing error rate, as calculated against a known mock community (positive control), indicates that the errors introduced during sequencing and analysis are within appropriate margins. However, although *Salmonella* Enteritidis was successfully cultured from the cecal contents of the control infected chickens and all the corresponding rarefaction curves approached asymptote, *Salmonella* was not detected in the 16S rRNA amplicon profiles. It appears that the levels of *Salmonella* colonizing the gut were below the limit of detection of this approach.

A mature, healthy gut microbiota is a significant hurdle to *Salmonella* colonization in both chicken and mouse models (13, 14, 72). It has been established that a protective effect against *Salmonella* colonization can be achieved by exposing young chicks (<7 days old) to the gut microbiota of mature birds (14). Deliberate endowment of colonization resistance to the chicks in intensive production systems has proved difficult as there is a risk of pathogen transfer from maternal birds, and although competitive exclusion products of mixed cocktails of bacteria are commercially available for seeding the chick gut (73, 74), they have faced steep regulatory hurdles in some territories. A recent study reported increases in the abundance of Bacteroidetes in the intestines of juvenile chickens after the donation of mature cecal microbiota, and the highlighted the bacterial taxa that readily colonize juvenile ceca to include *Megamonas* and *Phascolarctobacterium* (75), which were identified in the current study as responding to *S*. Enteritidis challenge with greater abundances evident on the jGOS diet. The juvenile birds inoculated with mature cecal contents also produced greater concentrations of cecal propionate and valerate (75). The introduction of taxa that can evolve SCFA in the intestines of juvenile birds to increase niche competition against pathogen colonisation could be advantageous but will require careful control to prevent inappropriate immune responses and facilitate the development of a productive microbiota (76). The deliberate manipulation of colonization resistance factors by the introduction of prebiotics is an attractive alternative to the direct introduction of a novel microbiota.

We have previously reported that GOS improves the growth rate of Ross 308 broiler chickens (25). In the results presented here, dietary GOS did not increase the body weight at 28 or 35 days, which is likely due to the absence of GOS in the finisher diet (24–35 days) (25). Previous studies that assessed the effect of supplementing feed with propionate on broiler performance noted a reduction in fat deposition and a reduction in average daily gain (77). However, *in situ* generation of propionate by cecal microbiota in response to *Salmonella* challenge represents a more focused and potentially more dynamic exposure that may be less likely to give rise to wider physiological effects. Outside the scope of this study, the association between *Salmonella* colonization in the absence of disease and growth performance merits further investigation.

Here, we report evidence that indicates that the process by which dietary GOS hastens *Salmonella* clearance is connected to the biochemical activities of specific bacterial taxa. We hypothesize that supplementary dietary GOS is catabolized to succinate in the cecal lumen, which cultivates increased populations of succinate utilizing bacteria. Succinate metabolism has been shown to have an important role in allowing *Salmonella* to colonize the mouse gut (78). The deleterious effect on *S*. Enteritidis colonization is therefore likely twofold: in the absence of inflammation, the increased concentrations of propionate and valerate are inimical to *Salmonella* growth, and increased competition for succinate presents an additional hurdle. However, the greatest relative abundance of Acidaminococcaceae and concentrations of propionate were detected after GOS supplementation of the feed had ceased implying that the early provision of GOS has a priming effect on the chicken cecal microbiome that enables the response to *Salmonella* colonization.

Supplementing the diet of juvenile chickens with GOS increases the proportion of a narrow category of bacterial taxa and confers a specific benefit to the host, i.e., hastening the eradication of intestinal *Salmonella* infection. Specific responses of the intestinal microbiota to GOS fulfill the prebiotic criteria described by Gibson et al. (16) and is an attractive approach as a safe and effective intervention against a key zoonotic, foodborne pathogen.

## MATERIALS AND METHODS

### Chickens

Commercial male Ross 308 broiler chicks were obtained as hatchlings (PD Hook, UK) and housed under strict biosecurity in a controlled environment operating at containment level 2 (https://www.hse.gov.uk/biosafety/assets/docs/management-containment-labs.pdf) at the University of Nottingham. Ambient temperature and relative humidity were controlled as dictated in the Code of Practice for the Housing and Care of Animals Bred, Supplied or Used for Scientific Purposes. Feeds were formulated on a least cost basis to meet the requirements set out in the Ross 308 Broiler Nutrition Specifications 2014 (Aviagen, UK) and prepared by Target Feeds Ltd. (Shropshire, UK). Throughout the trial, feed and water were provided *ad libitum*. In order to ensure that the birds were *Salmonella*-free upon arrival, the absorbent papers on which the birds had been transported were shredded and then added to Buffered Peptone Water (Oxoid, UK) for *Salmonella* enrichment as described below.

### Feeding regime

The feeding regime was as follows: the control diet group was sustained on a wheat-soybean meal-based feed provided as a starter mash at 0–10 days, grower pellets at 11–24 days, and finisher pellets at 25–35 days. The starter diet contained wheat (61.5% [wt/wt]), soya meal (31.1% [wt/wt]), soyabean oil (3.39% [wt/wt]), limestone (0.8% [wt/wt]), calcium phosphate (1.25% [wt/wt]), sodium bicarbonate (0.335% [wt/wt]), the enzymes phytase and xylanase (dosed according to the manufacturer’s instructions [DSM Nutritional Products Ltd., Switzerland]), and a vitamin mix containing salt, lysine HCl, DL-methionine, and threonine. The grower and finisher diets increased the wheat content at the expense of soya meal by 1 and 6% (wt/wt), respectively. Figure 1 summarizes the timings of changes in feed composition, *Salmonella* challenge, and sample collection. GOS was provided as Nutrabiotic GOS 64%, batch no. AQ8035 (Saputo Dairy, UK). Galactooligosaccharide preparations are manufactured by the β-galactosidase-catalyzed transglycosylation of lactose producing, in the case of batch AQ8035, glucose and galactose (22.4% [mass/mass] DM), lactose (10.1% [mass/mass] DM), and other disaccharides that are functionally included as galactooligosaccharides, and galactooligosaccharides [DP2 to DP8+ and typically of the form (gal)n-glc]. Nutrabiotic GOS 64% is produced as a feed grade syrup; batch AQ8035 has a dry matter concentration of 73.4% (mass/mass) and a total galactooligosaccharides concentration of 66.5% (mass/mass) DM. Starter feed was supplemented with 2.32% (wt/wt) GOS and isocaloric adjustments made in the wheat and soybean oil contents. The grower feed contained 1.16% GOS with the wheat and soybean oil contents adjusted accordingly. Nutrabiotic GOS was not added to the finisher feed. The final feeds were isocaloric with the same content of crude protein and Degussa poultry digestible amino acid. The feed formulations are listed in Table S3.

### Challenge trial

*Salmonella enterica* serovar Enteritidis P125109 (53) was selected as a challenge strain as the nalidixic acid resistance it exhibits facilitates enumeration through direct cultivation. The challenge trial was performed to determine the effects of *Salmonella* exposure on broiler chickens sustained on GOS-supplemented feed, where the experimental implementation and husbandry were conducted according to the ARRIVE guidelines (79). Upon arrival, chicks were weighed and randomly placed in pens of either five or six birds in one of two environmentally controlled containment rooms. Each room contained 14 independent pens with 7 pens per experimental group designated to receive either the control or GOS-supplemented feed. Samples were subsequently collected from a single chicken from each pen at each experimental timepoint. The coprophagous behavior of co-housed chickens means the birds are exposed to bacteria from each other’s intestinal content, and therefore, in this design, the pen is the independent experimental unit (*n* = 7). At 20 days old, chickens in one room were orally challenged with 4.6 × 10^8^ CFU *S*. Enteritidis suspended in 1 mL of Maximum Recovery Diluent (MRD; Oxoid, UK) or in the second room given an equivalent mock dose of sterile MRD. Following challenge, samples were collected at 2, 4, 8, and 15 days post-infection. The trial design is summarized in Fig. 1.

Live weights were recorded for the chickens selected at random from each pen for each timepoint and the birds euthanized by exposure to rising CO_2_ or parenteral barbiturate overdose followed by cervical dislocation in accord with Schedule 1 of the UK Animals (Scientific Procedures) Act. Sample tissues, cardiac blood, and intestinal contents were collected immediately post-mortem. Ileal tissues were sectioned from approximately 3 cm distal to Meckel’s diverticulum and cecal tissues from the distal tips of the ceca. Intestinal tissues were either flash frozen in liquid nitrogen for subsequent RNA isolation or preserved in 10% (wt/vol) neutral buffered formalin (Fisher Scientific, UK) for histological assessment. Approximately 1 g of cecal contents was aseptically collected from the distal end of each cecal pouch and flash frozen in liquid nitrogen before being stored at −80°C prior to genomic DNA isolation. A further 2–3 g sample of cecal contents was similarly collected from both ceca and combined in pre-weighed Universal vials before being stored on ice prior to *Salmonella* enumeration and SCFA analysis. Prior to incising the gut, the spleen and liver were aseptically excised with tissue samples preserved in 10% (wt/vol) neutral buffered formalin and the remainder placed in sterile polyethylene bags and stored on ice prior to *Salmonella* enumeration.

### *Salmonella* enumeration

Cecal contents were suspended at 0.1 g/mL and serially diluted 1:10 in MRD from which 100 µL aliquots over a range of dilutions were spread onto xylose lysine deoxycholate (XLD) agar (Oxoid, UK) modified with 1 µg/mL novobiocin (Sigma-Aldrich, UK) and 12.5 µg/mL nalidixic acid (Sigma-Aldrich). Cecal contents collected from each euthanized chicken were plated in triplicate and incubated at 37°C for 24 h prior to enumeration. Mean values of log_10_-transformed colony-forming units were used for statistical analysis. *Salmonella* was detected through enrichment by transferring ~0.1 g cecal contents or chick bedding papers to 10 mL buffered peptone water (BPW) that was then incubated at 37°C for 16–20 h before 0.1 mL volumes of the BPW suspension were dispensed onto modified semi-solid Rappaport-Vassiliadis (MSRV) agar (Oxoid) in triplicate. MSRV plates were incubated without inversion at 42°C for 24 h before any growth was sub-cultured to XLD plates (with no nalidixic acid or novobiocin added) and incubated at 37°C. Presumptive *Salmonella* colonies were confirmed with Poly O antiserum (Pro-Lab Diagnostics, UK). Liver and spleen tissues (1–5 g) were suspended 1:10 (wt/vol) in BPW and disrupted in a Seward Stomacher 80 (Seward Biomaster, UK) for 1 min on medium speed. Viable *Salmonella* present were recovered by direct plating of 0.1 mL of the tissue suspension onto modified XLD or through enrichment by incubating the remaining tissue suspension followed by isolation of any *Salmonella* present on MSRV. Incubation conditions and confirmation methods are as described above.

### 16S rRNA gene sequences and analysis

Total DNAs were isolated from cecal contents using a QIAcube HT apparatus and QIAamp PowerFecal Pro DNA kit (Qiagen, UK). Bacterial 16S rRNA gene sequences were PCR amplified using universal primers: 515f (5′ GTGCCAGCMGCCGCGGTAA 3′) and 806r (5′ GGACTACHVGGGTWTCTAAT 3′) designed to flank the hypervariable V4 region of the 16S rRNA genes (80). Amplicons were sequenced on the Illumina MiSeq platform (Illumina, USA) using 2 × 250 bp cycles according to the manufacturer’s instructions and the protocol described by Kozich et al. (81). Using the same protocol, no-template negative control amplicon libraries were prepared for both kit reagent and PCR batches and a positive control library was prepared from a defined mock community (ZymoBIOMICS Microbial Community DNA Standard, Zymo Research, USA). Mothur code to reproduce the analyses has been made available (see Data and code availability statement). All sequence data of the 16S rRNA genes were quality filtered and then clustered into OTUs in mothur v.1.48 (82) using the MiSeq as described by the package developers (81).

### Host gene expression analysis

Chicken intestinal total RNA was isolated from ileal and cecal tonsil tissues. Tissue biopsies were weighed out to 100 mg followed by homogenization with RLT lysis buffer supplied with the RNeasy kit (Qiagen, Germany) and 2.8 mm ceramic beads (MO BIO Laboratories Inc., Canada) in a TissueLyser II (Qiagen) bead mill. Total RNA was then extracted from lysates using the RNeasy kit and QIAcube HT (Qiagen). The quality of the extracted RNAs were analyzed using a TapeStation 2200 (Agilent, USA) by measuring the RNA integrity number. Reverse transcription of extracted RNA was carried out with the RT2 First Strand Kit (Qiagen) to synthesize the first-strand cDNA and remove any residual genomic DNA according to manufacturer’s instruction. The expression of host cytokine and chemokine genes were determined by quantitative polymerase chain reaction (qPCR) with RT2 SYBR Green qPCR Mastermix (Qiagen) in a Roche Diagnostics Light Cycler 480 instrument (Hoffmann La Roche AG, Switzerland). Primer sets were purchased as customized RT2 Profiler PCR Arrays (Qiagen) and verified to detect the transcripts for IFN-γ, IL-1β, IL-4, IL-6, IL-8L1, IL-8L2, IL-9, IL-10, IL-13, IL-15, IL-17A, IL-22, NOS2, STAT3, STAT4, STAT6, STAT5β, GATA3, and TGIF1 in chicken. The means of triplicate Ct values were used for analysis, where target genes Ct values were independently normalized to those of the housekeeping genes glyceraldehyde 3-phosphate dehydrogenase and 60S ribosomal protein L4. The normalized Ct values for the two housekeeping genes were in close agreement with an *R*^2^ of 0.94 across all determinations for cecal and ileal tissues.

### Histology

Tissue samples were fixed in a 10% formalin solution and dehydrated through a series of alcohol solutions, cleared in xylene, and embedded in paraffin wax (Microtechnical Services Ltd, Exeter, UK). Sections (3 to 5 µm thick) were prepared and stained with modified hematoxylin and eosin. Independent intestinal sections were stained with periodic acid-Schiff. The stained slides were scanned using a NanoZoomer Digital Pathology System (Hamamatsu, Welwyn Garden City, UK) at 40× resolution. The liver sections were examined for the infiltration of inflammatory cells around a central vein, whether porta hepatis or sinusoid. Villus heights and crypt depths of intestinal tissues were recorded from operator-blinded measurements of histological stained slides scanned for each tissue sample. Villus heights were measured from the tip of the villus to the crypt opening and the associated crypt depth was measured from the base of the crypt to the level of the crypt opening. The ratios of villus height to relative crypt depth (v/c ratio) were calculated from these measurements. Dimensions for 10 well-oriented villi per tissue sample of seven birds per experimental group at each sampling time were analyzed.

### Immunoglobin quantification

Anti-*Salmonella* IgY and IgA antibodies in chicken serum were detected by ELISA using the method of Withanage et al. (36) with the following modifications. *Salmonella* Enteritidis P125109 cells were subjected to three freeze-thaw cycles between room temperature and −80°C, followed by sonication on ice using 9 × 20 s bursts to break the cells. Unbroken cells were removed by centrifugation at 4,000 × *g* for 20 min at 4°C followed by filtration through a 0.2 µm filter. The protein concentration of the resultant soluble fraction was measured using Pierce BCA kit (Thermo Fisher, UK) and aliquots stored at −20°C. For ELISA, chicken serum samples were diluted 1:250 for IgY and 1:100 for IgA with phosphate buffered saline (PBS) containing Tween 20 (0.05%) (PBS-T) and 3% skimmed milk powder (SMP). Horseradish peroxidase-conjugated rabbit anti-chicken IgY (Sigma Aldrich) and goat anti-chicken (Abcam, UK) were diluted 1:5,000 in PBS-T and 3% SMP. The enzyme substrate used was 3,3′,5,5′-tetramethylbenzidine (Thermo Fisher) with the reaction stopped with 100 µL 2 M sulfuric acid after 30 min and absorbance read at 450 nm.

### SCFA quantification

Duplicate samples were analyzed for the SCFAs: acetic, (≥99%; Thermo Fisher), propionic, (99%; Thermo Fisher), butyric, (≥99%; Thermo Fisher), isobutyric, (≥99%; Thermo Fisher), 2-methylbutyric, (>95%; TCI UK Ltd., UK), valeric, (99%; Thermo Fisher), isovaleric, (99%; Thermo Fisher), hexanoic, (98%; TCI UK Ltd.), and lactic acid (90.2%; Thermo Fisher) by gas chromatography-mass spectrometry (GC-MS). Samples were prepared from weighed thawed cecal content material by sonication with the addition of 2-methylvaleric acid (>98%, Thermo Fisher) as internal standard. The SCFAs were converted to the ethyl ester and extracted into pentane (AR Grade; Thermo Fisher) along with the additions of the corresponding internal standards. The internal standards were separately synthesized as the deuterated ethyl ester for each SCFA, except for ethyl lactate D5-benzoate, the internal standard for lactic acid analysis. Sample SCFA concentrations were measured by GC-MS (Agilent 6890 + 5977 GC-MS; Agilent DB 5 MS UI, 15 m × 0.25 mm × 1.00 µm column; He carrier gas at 1.20 mL/min; using a defined oven temperature profile over a run time of 33.2 min; operated in selected ion monitoring [SIM] mode with 10 different SIM groups) with reference to a standard curve for each SCFA, with each calibrant being prepared using a procedure similar to that for the samples.

### Statistical analysis

Significant differences in the cecal burdens of *S.* Enteritidis between the challenged groups were tested using the Wilcoxon rank sum test with *P*-values ≤ 0.05, indicating significance. Significant differences in body weights at each timepoint was determined using the Kruskal-Wallis test. For those timepoints with a *P*-value below a significance threshold of 0.05, a subsequent pairwise Wilcox test with Benjamini-Hochberg adjustment for multiple comparisons was performed to test for significance between cohorts. For α-diversity as represented by the inverse Simpson index or Shannon entropy, statistical significance was determined using the Kruskal-Wallis test for each timepoint. For those timepoints with a *P*-value below a significance threshold of 0.05, a Dunn’s test of multiple comparisons with Benjamini-Hochberg adjustment was performed to determine significance between cohorts. Differences in expression for each pair of expression comparisons per gene per timepoint were determined using Student’s *t*-test with Benjamini-Hochberg adjustment. The ELISA results for the anti-*Salmonella* serum antibodies were analyzed using the Student’s *t*-test by comparing the *Salmonella*-colonized birds with the non-colonized controls on the same diet for each timepoint. For concentrations of each SCFA measured at each timepoint, statistical significance was determined using the Kruskal-Wallis test; for those SCFA with a *P*-value below a significance threshold of 0.05, a subsequent pairwise Wilcox test with false discovery rate correction was performed to determine significance between cohorts. Correlations between paired observations of SCFA concentration and bacterial abundance were estimated using Spearman’s ρ statistic in R. An analysis was made using concentrations of each SCFA measured against either bias-corrected or proportional taxonomic abundance. The *P*-values detected for each SCFA and each timepoint were adjusted using Benjamini-Hochberg correction. Statistical significance in villus length between cohorts was determined using the Tukey’s Honest Significant Difference method with adjustment for the multiple comparisons per timepoint. Differential abundance analysis of bacterial taxa was performed using the ALDEx2 (version 1.30.0) and ANCOM-BC (version 2.0.1) packages in R. ALDEx2 was performed using the aldex.clr modular approach. R code to reproduce both ALDEx2 and ANCOM-BC analysis has been made available on Github (https://github.com/PJRichards/richards_salmonella_gos).

All statistical analyses were performed in the R language (version 4.3.2) and all analyses were performed within a conda environment (https://docs.conda.io/en/latest/). The .yml recipe for the conda environment has been made available on Github (https://github.com/PJRichards/richards_salmonella_gos) alongside the R code to reproduce all analyses and figures. The design for Fig. 3C, D, and E was adapted from Wallen et al. (83).

## Data Availability

Amplicon sequence data have been deposited at NCBI and are publicly available under the accession number PRJNA972431. All original code has been deposited on Github (https://github.com/PJRichards/richards_salmonella_gos). Any additional information required to reanalyze the data reported in this paper is available on Github (https://github.com/PJRichards/richards_salmonella_gos) or is available on request.

## SUPPLEMENTAL MATERIAL

Figure S1 msystems.00754-24-s0001.pdf Rarefaction curves generated using re-sampling without replacement.

Figure S2 msystems.00754-24-s0002.pdf Alpha diversity described by Shannon entropy.

Figure S3 msystems.00754-24-s0003.pdf Effect of *S*. Enteritidis challenge on expression of genes connected to innate immune response

Figure S4 msystems.00754-24-s0004.pdf Salmonella serum antibodies.

Figure S5 msystems.00754-24-s0005.pdf Proportional OTU abundances and absolute concentration of the SCFA metabolome.

Table S1 msystems.00754-24-s0006.docx Prevalence of *S*. Enteritidis in liver and spleen tissues.

Table S2 msystems.00754-24-s0007.docx Abundance of discriminative OTUs observed in the no-template control.

Table S3 msystems.00754-24-s0008.docx GOS and control feed formulations.

ASM does not own the copyrights to Supplemental Material that may be linked to, or accessed through, an article. The authors have granted ASM a non-exclusive, world-wide license to publish the Supplemental Material files. Please contact the corresponding author directly for reuse.
