# Supplementary material for: Prebiotic galactooligosaccharide feed modifies the chicken gut microbiota to efficiently clear Salmonella
Source: mSystems. 2024 Jul 31;9(8):e00754-24. doi: 10.1128/msystems.00754-24 (PMC11334501; doi:10.1128/msystems.00754-24)
Supplement: Figure S1 — Rarefaction curves generated using re-sampling without replacement. [file msystems.00754-24-s0001.pdf]

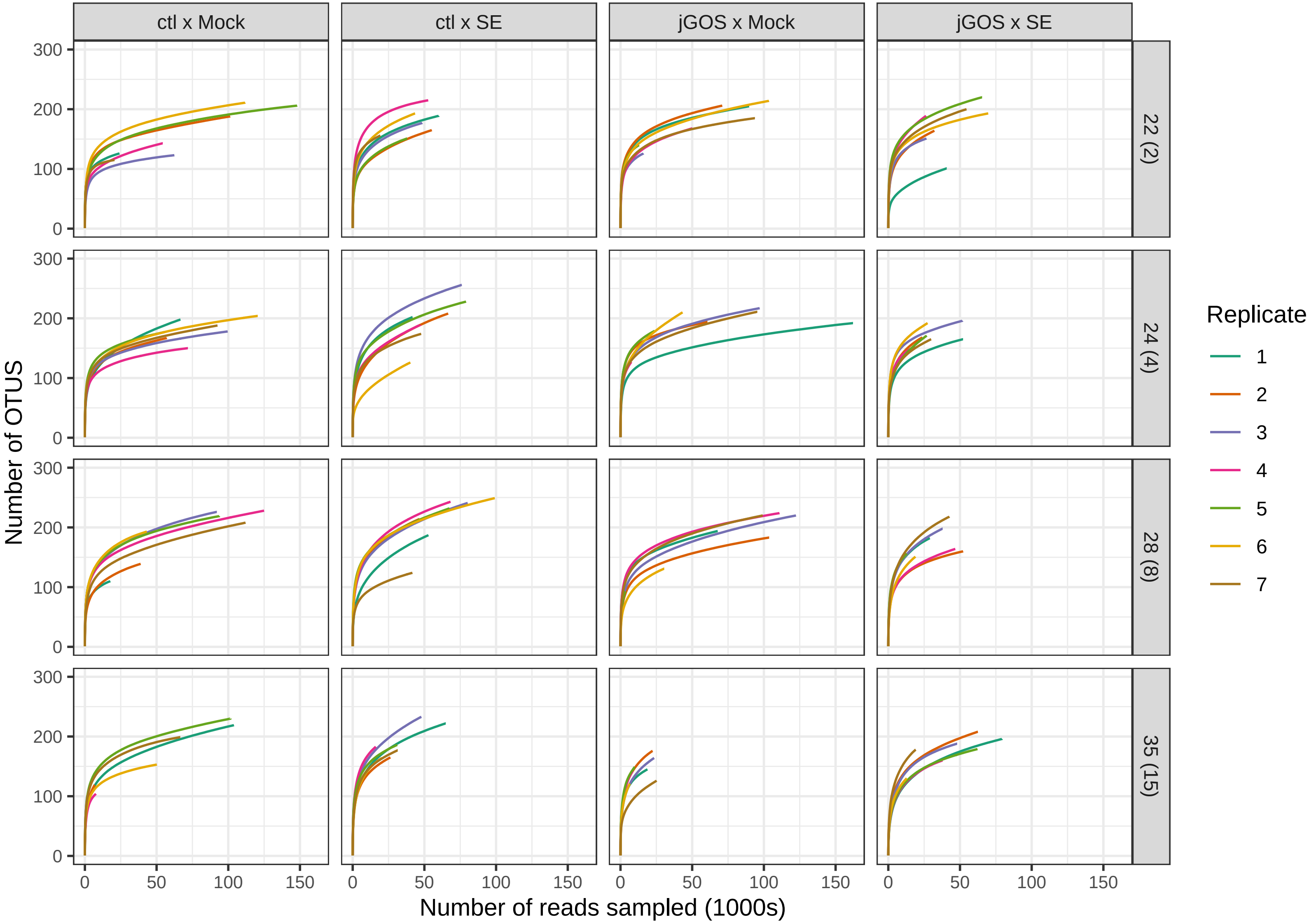

Figure S1. Sequencing effort for all communities. Rarefaction curves generated using re-sampling without replacement.
