# Supplementary material for: Prebiotic galactooligosaccharide feed modifies the chicken gut microbiota to efficiently clear Salmonella
Source: mSystems. 2024 Jul 31;9(8):e00754-24. doi: 10.1128/msystems.00754-24 (PMC11334501; doi:10.1128/msystems.00754-24)
Supplement: Figure S2 — Alpha diversity described by Shannon entropy. [file msystems.00754-24-s0002.pdf]

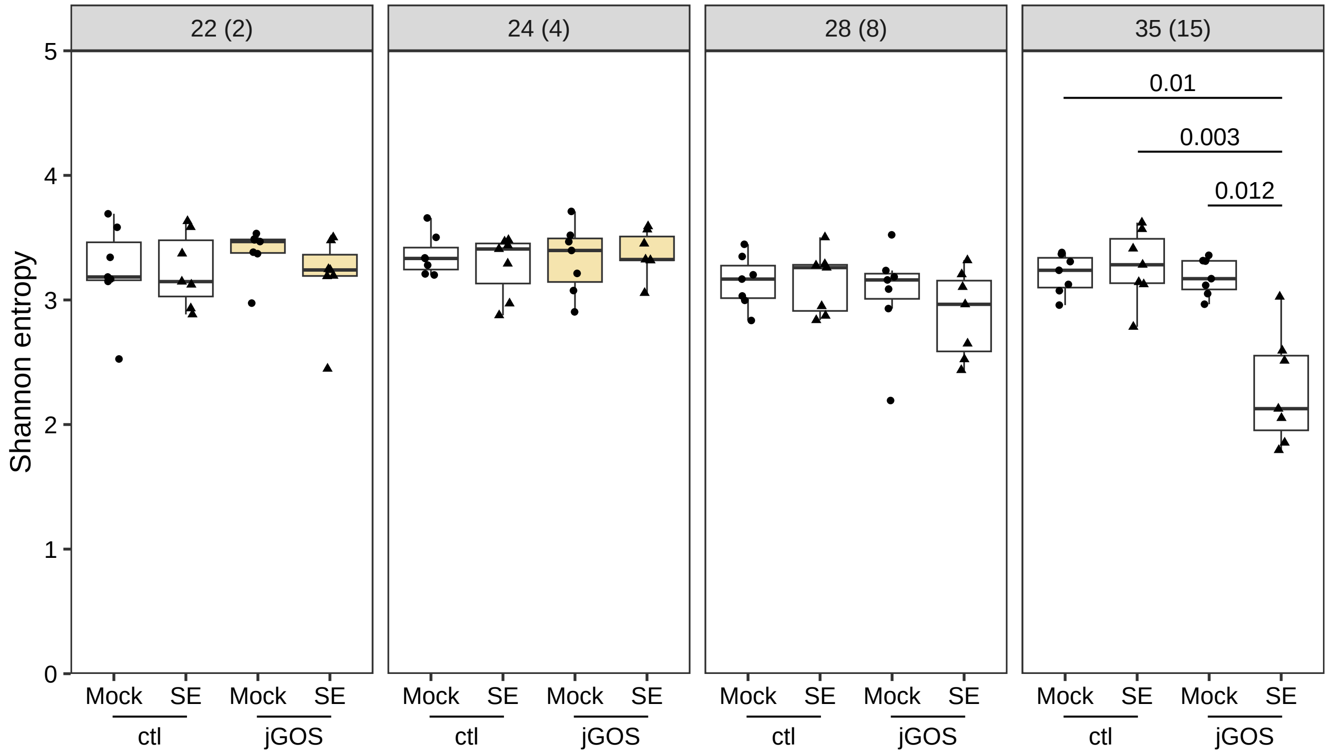

Figure S2. Supplementing feed with GOS in early life affects changes in the cecal microbiota caused by *Salmonella* challenge Alpha diversity is described using Shannon Entropy. Each data marker represents individual cecal community/bird collected from each independent pen (***n*** = 7). The shaded strip areas indicate the bird age in days followed by days post infection in parentheses. Statistical significance was determined using the Kruskal-Wallis test for inverse Simpson indices measured at each timepoint, for those timepoints with a ***p***-value below a significance threshold of 0.05, a Dunn's test of multiple comparisons with Benjamini-Hochberg adjustment was performed to determine significance between cohorts. The number above the bar indicates ***p***-value. Only ***p***-values < 0.05 are shown. The fill color indicates the diet at the time of sampling: unfilled, control feed without GOS; yellow, GOS supplemented feed.
