# Supplementary material for: Prebiotic galactooligosaccharide feed modifies the chicken gut microbiota to efficiently clear Salmonella
Source: mSystems. 2024 Jul 31;9(8):e00754-24. doi: 10.1128/msystems.00754-24 (PMC11334501; doi:10.1128/msystems.00754-24)
Supplement: Figure S3 — Effect of S. Enteritidis challenge on expression of genes connected to innate immune response [file msystems.00754-24-s0003.pdf]

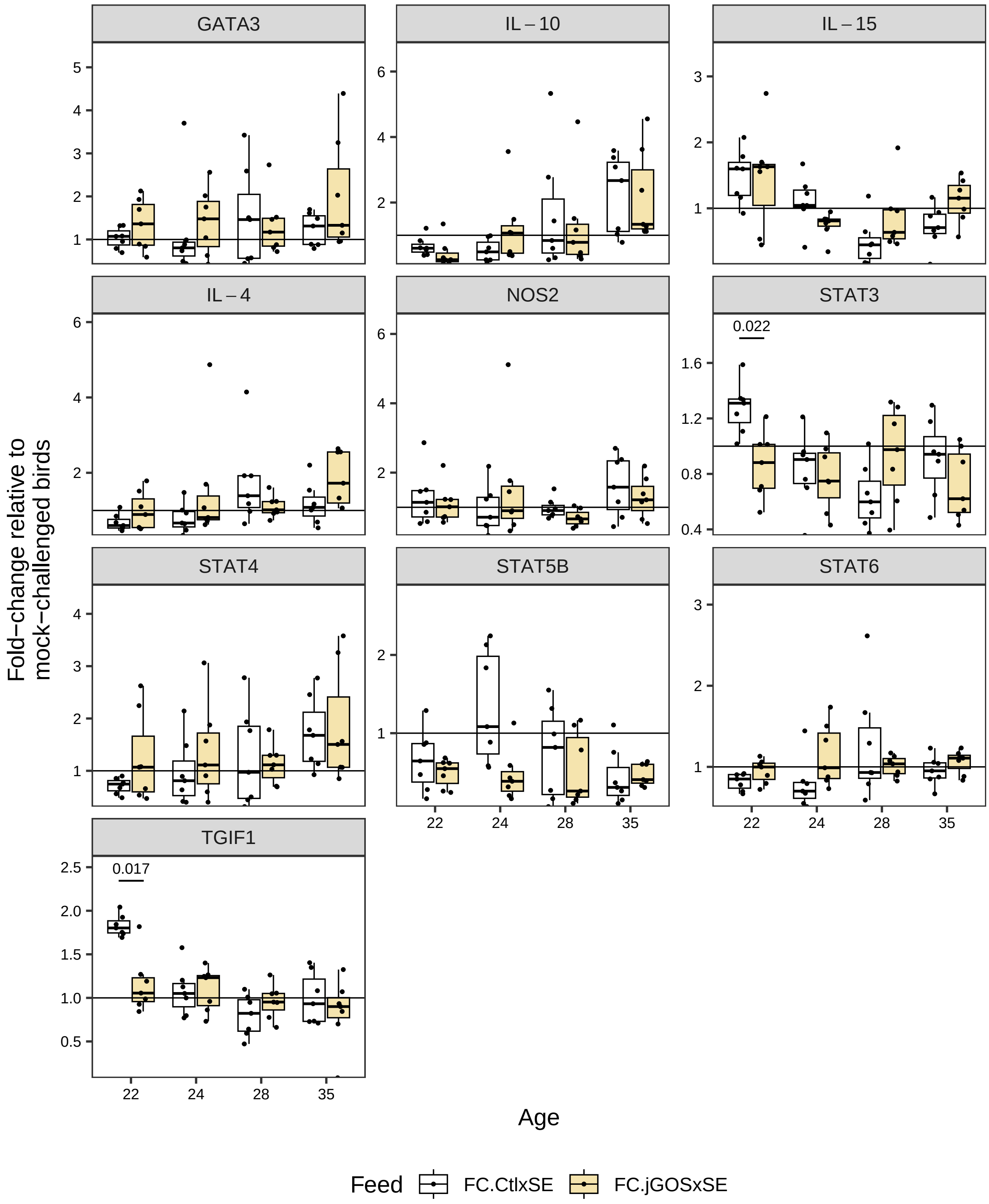

Effect of *S*. Enteritidis challenge on cecal expression of GATA3, IL-10, IL-15, IL-4, NOS2, STAT3, STAT4, STAT5B, STAT6 and TGIF1. Gene expression calculated as 2-ΔΔCt and presented as fold-change in ***Salmonella***-challenged chickens relative to the mean expression level of mock-challenged chickens for each feed group (Control or jGOS). Note that n = 7 for all groups presented here. The number above the bar indicates the adjusted p-value as determined using Students t-test for each pair of expression comparisons per gene per time point with FDR correction performed across each time point with the Benjamini-Hochberg method. Only p-values < 0.05 are shown. Shaded strips above each panel indicate the corresponding gene name.
