# Supplementary material for: Prebiotic galactooligosaccharide feed modifies the chicken gut microbiota to efficiently clear Salmonella
Source: mSystems. 2024 Jul 31;9(8):e00754-24. doi: 10.1128/msystems.00754-24 (PMC11334501; doi:10.1128/msystems.00754-24)
Supplement: Figure S4 — Salmonella serum antibodies. [file msystems.00754-24-s0004.pdf]

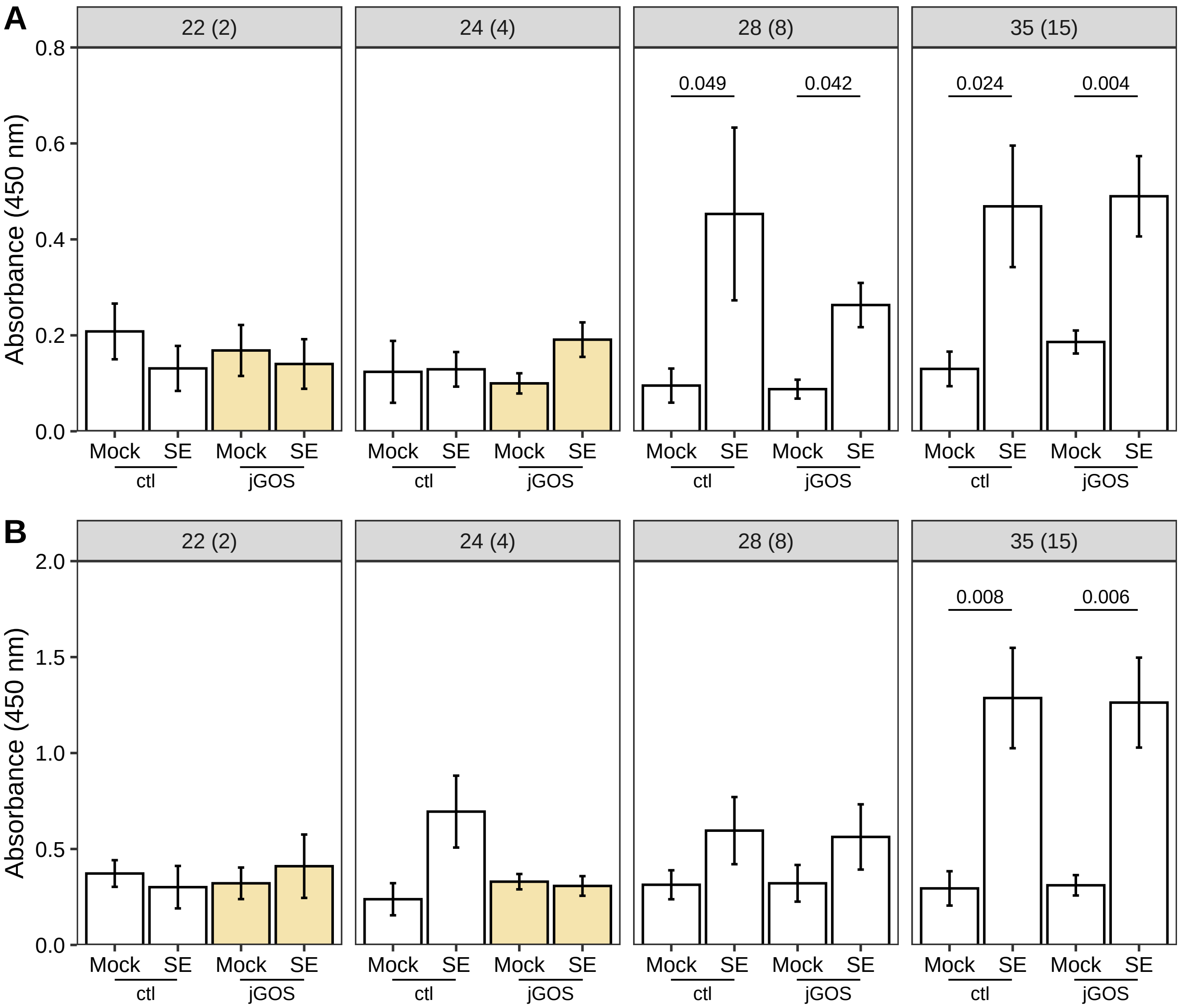

Figure S4. ELISA detection of *S*. Enteritidis specific serum antibodies from chickens infected with *S*. Enteritidis or mock-infected and raised on either a control or jGOS-supplemented diet. (A) IgY isotype anti-***Salmonella*** antibody detection (serum diluted 1:250). **(B)** IgA isotype anti-***Salmonella*** antibody detection (serum diluted 1:100). These data are recorded as the means of the optical density readings from the ELISA at 450 nm for seven birds per group at each time point (days of age). The error bars indicate the standard errors of the means. Bird age in days is shown on the x-axis with days post infection in parentheses. The number above the bar indicates the ***p***-values determined using the Student's t-test by comparing ***Salmonella*** colonized group with the non-colonized controls on the same diet for each timepoint.
