# Supplementary material for: Prebiotic galactooligosaccharide feed modifies the chicken gut microbiota to efficiently clear Salmonella
Source: mSystems. 2024 Jul 31;9(8):e00754-24. doi: 10.1128/msystems.00754-24 (PMC11334501; doi:10.1128/msystems.00754-24)
Supplement: Figure S5 — Proportional OTU abundances and absolute concentration of the SCFA metabolome. [file msystems.00754-24-s0005.pdf]

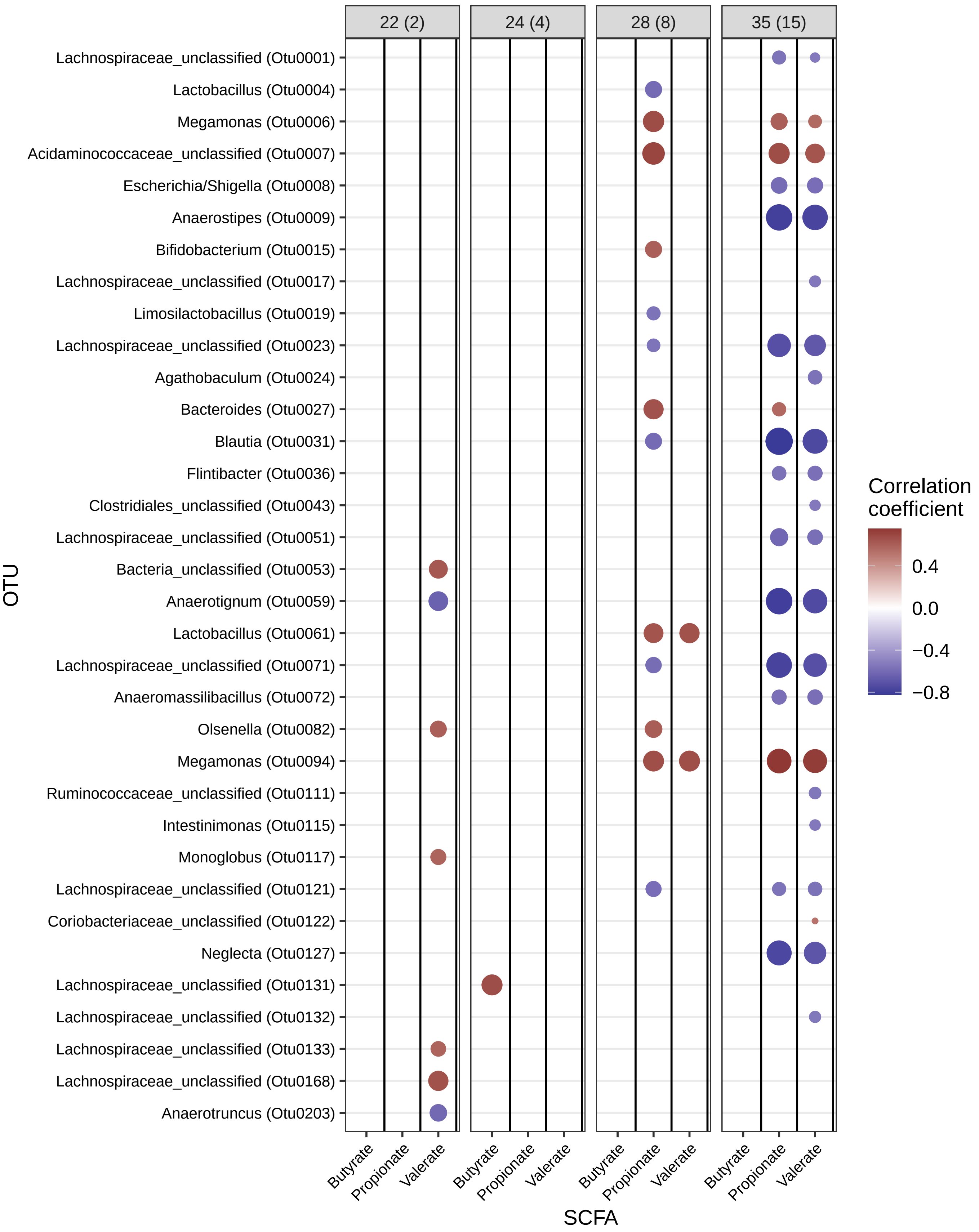

Figure S5. Correlation plot showing significant relationships between proportional OTU abundances and absolute concentration (ppm) of the SCFA metabolome. Correlation plot showing significant relationships between proportional OTU abundances and absolute concentration (ppm) of the SCFA metabolome. Both size and color of data marker indicate Spearman correlation coefficient with FDR correction for each SCFA at each timepoint. Filled data markers indicate significance (***p*** 0.05). Results are reported at OTU taxonomic levels. Due to failure to measure SCFA in the samples outlined in Figure 4, correlations were calculated on the basis of 27 measurements at 22 days, 28 measurements at 24, 28 measurements at 28 days and 26 measurements at 35 days.
