# Supplementary material for: Prebiotic galactooligosaccharide feed modifies the chicken gut microbiota to efficiently clear Salmonella
Source: mSystems. 2024 Jul 31;9(8):e00754-24. doi: 10.1128/msystems.00754-24 (PMC11334501; doi:10.1128/msystems.00754-24)
Supplement: Table S1 — Prevalence of S. Enteritidis in liver and spleen tissues. [file msystems.00754-24-s0006.docx]

|  |  | Liver | | Spleen | |
| --- | --- | --- | --- | --- | --- |
| Age | Group | - | + | - | + |
| 22 (2) | ctl x SE | 4 (57.1%) | 3 (42.9%) | 2 (28.6%) | 5 (71.4%) |
| 22 (2) | jGOS x SE | 0 (0%) | 7 (100%) | 0 (0%) | 7 (100%) |
| 24 (4) | ctl x SE | 0 (0%) | 7 (100%) | 0 (0%) | 7 (100%) |
| 24 (4) | jGOS x SE | 0 (0%) | 7 (100%) | 0 (0%) | 7 (100%) |
| 28 (8) | ctl x SE | 1 (14.3%) | 6 (85.7%) | 1 (14.3%) | 6 (85.7%) |
| 28 (8) | jGOS x SE | 1 (14.3%) | 6 (85.7%) | 0 (0%) | 7 (100%) |
| 35 (15) | ctl x SE | 5 (71.4%) | 2 (28.6%) | 4 (57.1%) | 3 (42.9%) |
| 35 (15) | jGOS x SE | 5 (71.4%) | 2 (28.6%) | 5 (71.4%) | 2 (28.6%) |

**Table S1. Prevalence of *Salmonella* Enteritidis in liver and spleen tissues following oral challenge at 20-days old.** The table shows the number of liver and spleen tissues collected from challenged chickens from which *Salmonella* could be isolated. The proportion of chickens is reported in parentheses.
