# Supplementary material for: Prebiotic galactooligosaccharide feed modifies the chicken gut microbiota to efficiently clear Salmonella
Source: mSystems. 2024 Jul 31;9(8):e00754-24. doi: 10.1128/msystems.00754-24 (PMC11334501; doi:10.1128/msystems.00754-24)
Supplement: Table S2 — Abundance of discriminative OTUs observed in the no-template control. [file msystems.00754-24-s0007.docx]

|  |  | OTU | | | | | | | |
| --- | --- | --- | --- | --- | --- | --- | --- | --- | --- |
| kitome | Sample name | Otu0006 | Otu0007 | Otu0015 | Otu0018 | Otu0027 | Otu0061 | Otu0063 | Otu0082 |
| 1 | l_kit_ctl_1 | 72 (95) | 0 (40) | 0 (41) | 0 (7) | 0 (64) | 0 (74) | 0 (4) | 0 (65) |
| 2 | l_kit_ctl_2 | 0 (23) | 0 (40) | 0 (41) | 0 (7) | 0 (64) | 0 (74) | 0 (4) | 0 (65) |
| 3 | l_kit_ctl_3 | 2 (48) | 0 (40) | 0 (41) | 0 (7) | 0 (64) | 0 (74) | 0 (4) | 0 (65) |

**Table S2. Abundance of discriminative OTUs observed in the no-template control kitome.** The table shows the number of reads that clustered with each discriminative OTU for each of the no-template control communities. Kitomes 1, 2 and 3 were prepared using different batches of DNA isolation kit reagents used for sample preparation. The figures in parenthesis indicate the rank of the read abundance for that OTU compared to all 112 communities surveyed, with 112 being the highest rank and 1 the lowest. Tied values are jointly assigned the highest rank.
