# Supplementary material for: Prebiotic galactooligosaccharide feed modifies the chicken gut microbiota to efficiently clear Salmonella
Source: mSystems. 2024 Jul 31;9(8):e00754-24. doi: 10.1128/msystems.00754-24 (PMC11334501; doi:10.1128/msystems.00754-24)
Supplement: Table S3 — GOS and control feed formulations. [file msystems.00754-24-s0008.docx]

| **FEED COMPONENT** | CONTROL:  ROSS 308 BROILER STARTER | CONTROL:  ROSS 308 BROILER GROWER | CONTROL: ROSS 308 BROILER FINISHER | GOS 2.32%:  ROSS 308 BROILER STARTER | GOS 1.16%:  ROSS 308 BROILER GROWER |
| --- | --- | --- | --- | --- | --- |
| WHEAT | 61.53 | 62.183 | 67.561 | 57.167 | 60.016 |
| EXT. HIPRO SOYA MEAL | 31.1 | 29.4 | 24.1 | 32.1 | 29.9 |
| LIMESTONE GRANULES | 0.80 | 0.60 | 0.60 | 0.80 | 0.60 |
| SOYABEAN OIL | 3.39 | 5.28 | 5.40 | 4.29 | 5.73 |
| LYSINE HCL | 0.437 | 0.253 | 0.238 | 0.416 | 0.243 |
| METHIONINE DL | 0.415 | 0.313 | 0.272 | 0.419 | 0.315 |
| DICALCIUM PHOSPHATE | 1.25 | 1.00 | 0.87 | 1.27 | 1.00 |
| SODIUM BICARBONATE | 0.335 | 0.251 | 0.247 | 0.321 | 0.244 |
| SALT | 0.110 | 0.170 | 0.170 | 0.120 | 0.170 |
| THREONINE | 0.183 | 0.100 | 0.092 | 0.177 | 0.097 |
| TM - Blank Premix for Broiler Formulation | 0.400 | 0.400 | 0.400 | 0.400 | 0.400 |
| **Nutrabiotic**® **GOS** | **0.000** | **0.000** | **0.000** | **2.320** | **1.160** |
| RONOZYME P5000 (CT) | 0.030 | 0.030 | 0.030 | 0.030 | 0.030 |
| Ronozyme WX (Xyl) | 0.020 | 0.020 | 0.020 | 0.020 | 0.020 |

**Table S3 GOS and control feed formulations**. Feed formulations for starter (0-10 days of age), grower (11-24 days of age) and finisher (25-35 days of age) diets.
